# Genetically Encoded Voltage Indicators in Circulation Research

**DOI:** 10.3390/ijms160921626

**Published:** 2015-09-08

**Authors:** Lars Kaestner, Qinghai Tian, Elisabeth Kaiser, Wenying Xian, Andreas Müller, Martin Oberhofer, Sandra Ruppenthal, Daniel Sinnecker, Hidekazu Tsutsui, Atsushi Miyawaki, Alessandra Moretti, Peter Lipp

**Affiliations:** 1 Research Centre for Molecular Imaging and Screening, Institute for Molecular Cell Biology, Saarland University, Homburg/Saar 66421, Germany; E-Mails: qinghai.tian@uks.eu (Q.T.); Elisabeth.Kaiser@uks.eu (E.K.); Wenying.Xian@uks.eu (W.X.); martin.oberhofer@gmx.de (M.O.); Sandra.Ruppenthal@uks.eu (S.R.); Peter.Lipp@uks.eu (P.L.); 2 Department of Diagnostic and Interventional Radiology, Saarland University Medical Center, Homburg/Saar 66421, Germany; E-Mail: Andreas.Mueller@uniklinikum-saarland.de; 3 I. Medizinische Klinik und Poliklinik, Klinikum rechts der Isar der Technischen Universität München, Munich 81675, Germany; E-Mails: sinnecker@tum.de (D.S.); amoretti@mytum.de (A.M.); 4 Department of Material Science, JAIST, Nomi, Ishikawa 923-1292, Japan; E-Mail: tsutsui@jaist.ac.jp; 5 Cell Function Dynamics, Brain Science Institute, RIKEN, Wako 351-0192, Japan; E-Mail: matsushi@brain.riken.go.jp; 6 DZHK (German Centre for Cardiovascular Research)-partner site Munich Heart Alliance, Munich, Germany

**Keywords:** Genetically Encoded Voltage Indicators (GEVI), membrane potential, cardiomyocyte, action potential

## Abstract

Membrane potentials display the cellular status of non-excitable cells and mediate communication between excitable cells via action potentials. The use of genetically encoded biosensors employing fluorescent proteins allows a non-invasive biocompatible way to read out the membrane potential in cardiac myocytes and other cells of the circulation system. Although the approaches to design such biosensors date back to the time when the first fluorescent-protein based Förster Resonance Energy Transfer (FRET) sensors were constructed, it took 15 years before reliable sensors became readily available. Here, we review different developments of genetically encoded membrane potential sensors. Furthermore, it is shown how such sensors can be used in pharmacological screening applications as well as in circulation related basic biomedical research. Potentials and limitations will be discussed and perspectives of possible future developments will be provided.

## 1. Measuring Membrane Potentials—Principles and Properties

The membrane potential, especially the action potential of muscles and nerves, has been measured since the middle of the 19th century [1,2,3], using metal electrodes. The transition to the cellular level started in the 1930s [4] with the development of the voltage clamp approach, which was greatly improved by the introduction of the patch-clamp technique [5,6]. Patch-clamp is still regarded as the Gold-standard for cellular electrophysiology. The big advantage of this approach is that the entire cell can be controlled, *i.e*., clamped to a given potential (voltage-clamp mode) to monitor the membrane current or alternatively to a given current (current-clamp mode) to monitor the membrane potential. However, this method bears some considerable disadvantages: (i) cells need to be mechanically disturbed by the glass pipette; (ii) spatial information is limited to a cell or a patch of membrane without simultaneous recording of the potential distribution across the cell membrane; and (iii) moving cells, like cardiomyocytes within an intact beating heart can not be characterized. All these limitations can be overcome by using contact-free optical read-outs. All optical sensors for investigations of the membrane potential developed so far, independent of whether they rely on small molecule dyes or genetically encoded chromophores, are based—directly or indirectly—on membrane potential-induced changes of fluorescent properties. Fluorescence read-out has general advantages over absorption detection [7]. Nevertheless, for a high spatial and temporal resolution (≤1 ms) maintained over a recording duration of minutes, the number of emitted photons becomes the limiting factor. An alternative is non-fluorescent optical recordings, as it was shown for dark field microscopy based on abnormal dispersion [8]. This study depicted that the dark field optical signal was linear proportional to the change in membrane potential.

Small molecule dyes have a number of advantageous properties, but suffer from the unspecific staining of all cell types. A possible alternative is genetic targeting using tissue specific promotors. In addition to the fluorescent protein based voltage sensors (see below), it is worthwhile to mention the combination of genetic targeting and conventional organic chromophores [9]. In a proof of principle report, it was described that a membrane targeted phosphatase was able to cleave the hydrophilic phosphate group of a precursor dye, leading to a membrane bound voltage sensitive dye [10]. A further hybrid approach utilized the expression of a membrane bound GFP as Förster Resonance Energy Transfer (FRET) donor in combination with dipicrylamine (DPA), a synthetic voltage sensing molecule, as FRET acceptor [11]. This hybrid approach has the advantage of genetic targeting but suffers from the common disadvantages of small molecule dyes such as the limitations for long-term observation. There are controversial reports about the usability of this construct. While DiFranco and colleagues applied it successfully in the transverse tubules in mouse skeletal muscle fibers [12] and recently Ghitani *et al.* [13] reported imaging of spikes and synaptic potentials in single neurons, Sjulson and Miesenböck showed that—due to DPA-induced increase in membrane capacitance—it was not possible to detect action potentials in the Drosophila antennal lobe [14].

Optical membrane potential sensors, whether small molecules, genetically encoded, or combinations thereof, share the property of reporting primarily membrane potential changes and not an absolute voltage. Ratiometric sensors allow in principle a calibration to absolute voltage, but obstacles like different bleaching of donor and acceptor in FRET based sensors render such procedures difficult. Another approach is to consider time domain based changes of photopysical properties like fluorescence lifetime imaging. In line with such considerations, a special microbial rhodopsin was engineered, where the temporal dynamics of the fluorescence was read out in pump-probe experiments reaching a voltage accuracy of 10 mV [15]. Although a quantitative calibration for particular indicators is possible, it has technical challenges and is therefore rarely used.

## 2. Approaches of Genetically Encoded Voltage Indicators

Genetically encoded membrane potential sensors, fluorescent protein based voltage sensors or optogenetic voltage reporters are different synonyms for the same kind of membrane potential probes that are termed GEVIs (Genetically Encoded Voltage Indicators) throughout this review. Beside all varieties throughout the genesis of GEVIs, they all share voltage sensing domains that are based on (or part of) an integral membrane protein, which makes GEVIs a nice example for the Special Issue “Membrane Protein Based Biosensors”.

### 2.1. GEVI (Genetically Encoded Voltage Indicators) Based on Voltage Sensitive Conformational Changes

The first voltage sensors solely comprising genetically encoded proteins (called FlaSh) comprised a wtGFP fused to the C-terminus of the *Drosophila* Shaker K^+^-channel [16]. Later, FlaSh was improved for kinetics and voltage range [17]. The second GEVI generated independently was based on the fourth transmembrane segment (S4) of the voltage gated K^+^ channel K_v_2.1 coupled to a CFP/YFP FRET pair in sequence and was named VSFP1 [18]. This sensor was followed by a circular permutated version of the fluorescent protein [19]. The third GEVI type was called SPARC and comprised a GFP fused between domains I and II of the rat skeletal muscle Na^+^ channel [20]. These three GEVIs lack distinct membrane localization [21]. In addition, they all displayed only modest fluorescence changes (0.5%–5%) for a membrane potential change of 100 mV [16,18,20]. The combination of both properties made them fail in biological applications. A new generation of GEVIs comprised self-contained voltage sensor domains, such as the voltage sensing domain of the *Ciona intestinalis* Voltage Sensor-containing Phosphatase (Ci-VSP) [22], or voltage sensor domain only proteins [23].

**Figure 1:**
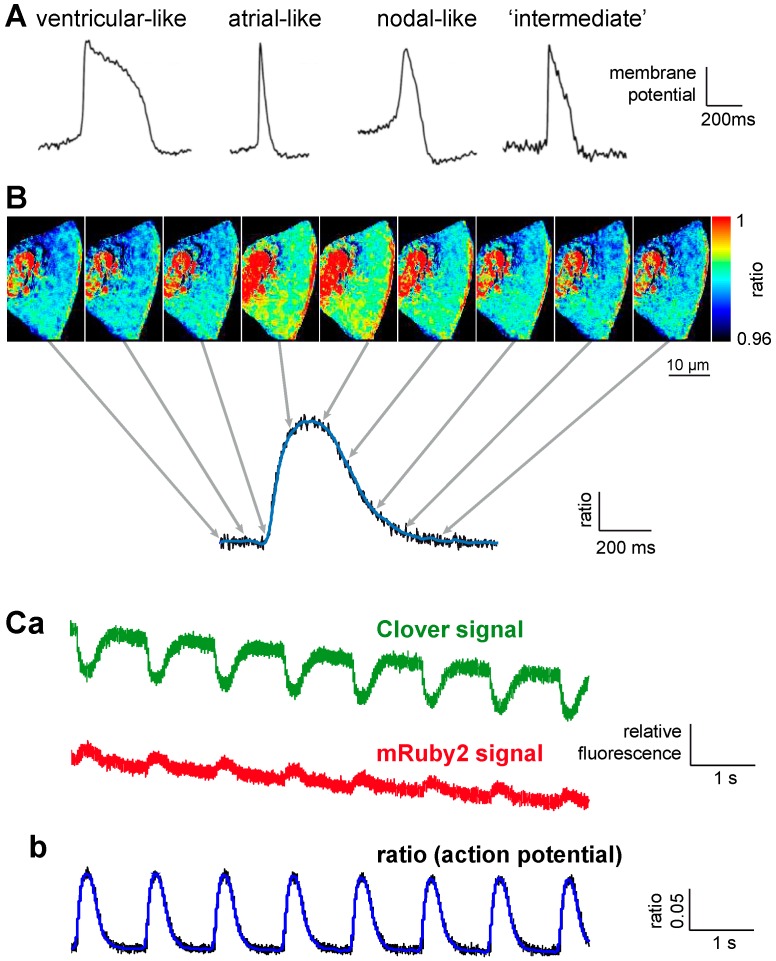
Voltage-Sensitive Fluorescent Protein Clover-mRuby2 (VSFP-CR) for phenotyping stem cell derived cardiomyocytes. (**A**) Overview of optically recorded (Di-8-ANEPPS) reference action potential phenotypes from induced pluripotent stem cell derived cardiomyocytes. This panel is reproduced from [28], with permission from John Wiley & Sons; (**B**) Recorded image series of a human stem cell derived cardiomyocyte expressing VSFP-CR (Lentiviral gene transfer). The images are snapshots every 100 ms of a time series recorded at 500 frames per second using a scientific Complementary Metal-Oxide-Semiconductor (sCMOS) camera and point to the time course of the recorded action potential. Considering the temporal response of the GEVI (Genetically Encoded Voltage Indicators), the example shows most alikeness with an “intermediate” action potential with a tendency to the ventricular phenotype. Overlay of the raw ratio trace (black) and a smoothed trace (blue); (**C**) Original and processed traces of a train of recorded action potentials of an electrically paced (1 Hz) stem cell derived cardiomyocyte. (**a**) Raw traces of the spectral channels for Clover and mRuby2; (**b**) Overlay of the raw ratio trace (black) and a smoothed trace (blue).

The Ci-VSP domain was chosen by two groups of the same institution (Brain Science Institute, RIKEN, Japan) that independently developed what is now termed VSFP2.x [24] and Mermaid [25]. Based on the VSFP2.1 design, further developments were undertaken. Linker optimization led to VSFP2.3. In a study using spectrally resolved data VSFP2.3 and Mermaid displayed similar ratio changes of around 13% per 100 mV potential change under seemingly similar conditions [26]. Based on VSFP2.3 linked to a pair of fluorescent proteins improved for FRET (Clover and mRuby2), an enhanced sensor termed VSFP-CR was introduced (Figure 1) [27]. Selected basic biophysical properties of the most popular of these and the following described GEVIs with an emphasis on circulation research are summarized in Table 1.

**Table 1:** Overview of the most popular Genetically Encoded Voltage Indicators (GEVIs), their properties and application in circulation research.

#	GEVI	Voltage Sensing Principle	FRET Pair (1–3, 8) Fluorescent Protein (4–7)	Principle Design and Operation with Permission from Elsevier [29]	*∆R/R* per 100 mV (1–3) *∆F/F* per 100 mV (4–8)	Detection Range	Temporal Response (on); Jump from –70 mV to at least +30 mV	Application in Circulation Research/Comments
1	VSFP2.3 [30]	conformational change by phosphatase of sea squirt (*Ciona intestinalis*) [22]	mCerulian (CFP) and Citrine (YFP) [30]	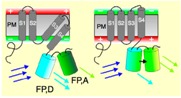	13.3% ± 3.4% [26], 10% ± 1% [27]	half activation ~–40 mV [27]	biexponential 2.5 ± 0.5 and 25 ± 3 ms −70 to +60 mV and 35 °C [26]	optical mapping in transgenic mouse heart [31]
2	Mermaid [25]		Umi Kinoko (mUKG) and the monomeric Kusabira Orange (mKOκ) [25]		12.9 ± 4.8% [26]	half activation ~–40 mV [25]	biexponential 2.5 ± 0.5 ms (23% ± 5%) and 25 ± 3 ms at 35 °C [26]	cardiotoxicity screens *in vivo* (zebrafish) [32] and in isolated cardiac myocytes (rat) [33]; optical mapping in transgenic mouse heart (this paper)
3	VSFP-CR [27]		Clover and mRuby2 [27]		13 ± 1% [27]	half activation ~–40 mV [27]	biexponential 5.4 ± 0.8 and 59.5 ± 5.5 ms at 20 °C [27]	measurements in stem cell derived cardiomyocytes for phenotyping (this paper)
4	ArcLight [34]		super ecliptic pH luorin (A227D) GFP [35]	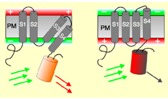	~32% [36]	half activation ~–25 mV [34]	biexponential ~17.4 ms (~39%) and ~123 ms at 23 °C [36]	stem cell derived cardiomyocyte phenotyping [37]
5	ASAP1 [38]	chicken (*Gallus gallus*) voltage-sensitive phosphatase [38]	circularly permutated GFP	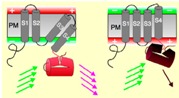	~29% [39]	–120 to −50 mV superlinear and –50 to 50 mV linear response [38]	biexponential 2.1 ± 0.2 ms (60.2%± 1.2%) and 71.5 ± 1.6 ms [38]	to be done (t.b.d.)
6	Arch (D95N) [40]	microbial rhodopsin proton pumps	modified Archae-rhodopsin 3 [40]	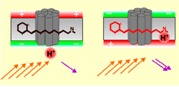	~40% [36]	–150 to +150 mV almost linear response [40]	biexponential < 0.5 ms (~20%) and ~41 ms [40]	mapping of membrane potential in transgenic zebrafish heart [41]
7	QuasAr2 [36]		modified Archae-rhodopsin 3 [36]		90% ± 2% [36]	–100 to +50 mV almost linear response [36]	biexponential 1.2 ± 0.1 ms (68%) and 11.8 ± 1.5 ms; similar on rising and falling edge [36]	t.b.d./most bathochromic GEVI (exitation 590 nm, emission 715 nm); although high sensitivity, fluorescence intensity is ∼50-fold dimer than GFP [39]
8	MacQ [42]	voltage induced shifts in the absorption spectrum of *Leptosphaeria maculans* rhodopsin results in quenching of the attached mCitrine or mOrange2 [42], although FRET is happening, only the intensity change of the donor is measured		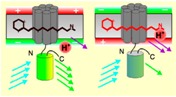	~20% [42]	–100 to 0 mV almost linear response, leveling out at 0 mV [42]	biexponential 2.8 ± 0.2 ms (74% ± 2%) and 71 ± 3 ms (26% ± 2%) for mCitrine and 2.9 ± 0.1 ms (96% ± 1%) and 115 ± 10 ms (4% ± 1%) for mOrange2 [42]	t.b.d./based on the same principle a palette of multicolored GEVI have been introduced [39]

A further line of development explored alterations in the fluorescent entities: circular permutated fluorescent proteins [43] and red-shifted variants [44] with positive proof of principles but moderate intensity changes were generated. Another approach, in contrast to previous designs, was to sandwich the voltage sensing domain (of VSFP2.x) between the two fluorescent proteins resulting in VSFP “Butterfly” [45]. Using the two fluorescent proteins mCitrine and mKate yielded a membrane probe that allowed imaging of electrical responses of the somatosensory cortex in head fixed mice as a proof of principle *in vivo* [45].

All GEVIs so far share the property of a fast and a slow kinetic response component. While the fast component results from sensing currents within the voltage sensing domain, the slow component is a consequence of the voltage-dependent conformational change in the probe [46]. The Knöpfel group performed seminal work in linker and fluorescent read-out optimization and introduced a novel probe named VSFP3.1 [30]. This construct was characterized by a dramatic shift of the slow sensing component towards faster read-out kinetics. This approach based on a response of the voltage sensing domain in the absence of major conformational changes of the fluorescence components and thus no changes in intramolecular FRET. These properties resulted in intensity changes of approximately 0.5% per 100 mV potential change [30]. When considering the overall properties of VSFP3.1, it appears to be of restricted use. Similar to this, other approaches employing voltage sensing domains of voltage-gated phosphatases of other species, in particular starlet sea anemone (*Nematostella vectensis*) and zebrafish (*Danio rerio*), also resulted in sensors with a fast kinetic response (2–5 ms) but with rather small intensity changes (0.3% per 100 mV voltage change) [47].

Based on Mermaid, an improved GEVI was designed using a similar rational as for the VSFP3.x probes [34] but taking super ecliptic pHluorin [35] as the fluorescent protein. This sensor was named ArcLight and displayed a large fluorescence response of more than 30% per 100 mV voltage change [34,36]. For ArcLight, a replacement of the voltage sensing domain from that of the sea squirt to the one from chicken (*Gallus gallus*) and zebrafish (*Danio rerio*) was reported to improve the temporal response, but at the expense of the response amplitude [48].

According to an initial report, the Accelerated Sensor of Action Potentials (ASAP1) is currently the best non-ratiometric GEVI in this group of voltage sensitive fluorescent proteins [38]. It is based on the voltage-sensitive phosphatase of chicken (*Gallus gallus*) and displays around 29% fluorescence change per 100 mV voltage change [39]. In addition, the kinetic was also advantageous, the activation response of the fast component of 2.1 ± 0.2 ms represented approximately 60% of the total signal amplitude (Table 1) [38].

### 2.2. Microbial Opsin-Based GEVIs

A completely different concept for GEVI design is based on the use of microbial opsins [49] and resulted in the development of sensors named PROBS and Arch [40,49]. The latter one is derived from the rhodopsin protein, Archaerhodopsin 3 [40]. Microbial opsins bind retinal, a vitamin A-related organic chromophore, and have evolved naturally to function as transducers of light into cellular signals. These proteins are known as tools for optogenetic manipulation [50]. The natural occurring relationship between light and voltage can be reversed, so that membrane voltage changes are reported as an optical signal. In the initial construct of Arch, the light required for imaging activated a proton current resulting in a contra productive change of the membrane potential. Although a point mutation (D95N) abolished Archs’ capacity to elicit light-driven currents, it also impaired the temporal response [40].

The microbial opsin-based GEVIs were improved ever since leading to new versions of Arch, like Arch-EEN and Arch-EES [51], Archer1 and Archer2 [52] and the QuasAr’s (QuasAr1 and QuasAr2) [36]. QuasAr2 displays a substantial change in fluorescence per 100 mV change of membrane potential of approximately 90% and an activation response of the fast component of 1.2 ± 0.1 ms that reflects approximately 68% of the response (Table 1) [36]. Although QuasAr2 has a high dynamic response, its overall fluorescence intensity is 30- to 80-fold dimmer than GFP [39].

The combination of fluorescent proteins with a fungal rhodopsin (*Leptosphaeria maculans*) to perform FRET resulted in the development of MacQ-GEVIs with a good responsiveness of around 20% per 100 mV of voltage change and an activation response of the fast component of 2.2 ± 0.2 ms representing approximately 74% of the total signal (Table 1) [42]. A very similar strategy was performed combining QuasAr2 with various fluorescent proteins from eGFP to mKate2 [39].

### 2.3. Sensing Non-Linear Optical Properties of Fluorescent Proteins

All previously described approaches using genetically encoded voltage sensors are based on native voltage sensing proteins that functionally rely on protonation or conformational changes, such as voltage dependent protonation of the retinal Schiff base or voltage dependent phosphatases. Their mechanical action towards conformational changes in the sensing domains induce steric alterations in the fluorescent proteins that are utilized to provoke and subsequently measure changes in fluorescence intensity. A different approach would be to explore possible interactions between the membrane potential and the chromophore itself. The Stark effect caused by electric field changes is used in small molecular dye-based voltage sensors, e.g., [53]. However, for chromophores of fluorescent proteins this effect is too small to be detected by fluorescence microscopy. This highlights other properties of chromophores that have hardly been appreciated in the development of biosensors in general. These are the non-linear properties allowing the employment of second harmonic generation (SHG) in response to femtosecond pulsed infrared light. The general concept [54,55] and initial attempts [55,56] are summarized in [57] but they were not explored further.

## 3. Examples of GEVIs in Circulation Research

With the development of the sCMOS technology camera acquisition rates in combination with high quantum efficiencies (up to 0.7 for front illuminated sensors) have reached a level that allowed the transition from photometric measurements of individual cells to area detectors [58]. The latter detectors enable the simultaneous recording of cell populations in combination with good subcellular resolution [59]. Although GEVIs seem to be much more popular in neurosciences compared to circulation research [60], we identified three major heart related applications, which are detailed below. In addition to cardiac myocytes, other (non-excitable) cells of the circulation show membrane potential changes, like T-cells when activated [61], red blood cells under volume regulation [62] or endothelial cells of vessels under inflammation [63]. However, these rather moderate changes in membrane potential were not compatible with rather limited intensity changes of many of the GEVIs. However, latest developments [36,38,39,42] may enable further applications in the above-mentioned examples.

### 3.1. Cardiotoxicity Screens

Conceptual studies of cardiotoxicity screens based on GEVI have been performed with “Mermaid”, a sensor introduced in 2008 [25]. Mermaid displayed relative ratio changes around 13% per 100 mV membrane change (measured between the membrane potentials of −80 and +20 mV) [25] and therefore compares well with small molecule dyes such as RH-237 or di-8-ANEPPS [64]. We even noticed a 25% higher change of the relative fluorescence ratio compared to the ratiometric read-out mode of di-8-ANEPPS [65]. We have to note that these values refer to a simple ratio of the two spectral channels allocated to the FRET donor and acceptor. Calculation of the real FRET efficiency or the apparent FRET efficiency [66] has not been achieved yet, because alternating dual excitation has not yet met the necessary temporal resolution. However, in adult cardiomyocytes, pharmaceutical prolongation of the action potential could be detected readily [33]. This prolongation of the action potential duration can be regarded as a cellular equivalent of the QT-interval prolongation in the ECG, which is a pro-arrythmogenic indicator [67]. Thus, optical measurements of action potentials in cardiomyocytes expressing a GEVI allow for pharmacological safety screens, as shown in pilot studies [33,65].

Furthermore, a transgenic zebrafish (*Danio rerio*) expressing Mermaid under the control of a cardiac specific promotor [32] demonstrated the first *in vivo* imaging of voltage dynamics in a whole heart. Although this study is eminent for having the first optical *in vivo* potential recordings of the entire heart, transfer from zebra fish cardiac physiology to relevant tasks of mammalian and human circulation research is rather equivocal.

### 3.2. Stem Cell Derived Cardiomyocyte Phenotyping

Differentiating cardiomyocytes from embryonic or induced pluripotent stem cells (iPS-cells) is becoming increasingly popular with a wide variety of applications [68,69]. However, the stem cell derived cardiomyocytes contain a mixture of different phenotypes, like ventricular myocytes, atrial myocytes or myocytes of the conduction system. For the experimental design as well as for further differentiation, it is desirable to purify or just identify a particular subtype of cardiomyocytes. All subtypes have a different gene expression, but are morphologically indistinguishable. A method to discriminate the cell type is the shape of their action potential, which is characteristic for the subtypes mentioned above [28]. An elegant way to measure such an action potential is by means of a GEVI, as shown for ArcLight expressed in cardiomyocytes from human embryonic stem cells [37].

Although ArcLight, which was introduced in 2012 [34], is not a ratiometric GEVI, it could resemble the action potential shape and thus allow for a phenotyping of the stem cell derived cardiomyocytes [37]. Further advancements might be possible with the introduction of novel ratiometric GEVIs like VSFP-CR that allows lentivirus-mediated expression in induced pluripotent stem cell derived cardiac myocytes, as seen in Figure 1.

### 3.3. Optical Mapping in Transgenic Heart

Mapping of action potentials in excised hearts is a popular method for exploring pathophysiological processes preferentially in animal models. Electrode arrays have been used for such purpose, but they have a limited spatial resolution [70]. An alternative is optical mapping that was so far performed with small molecule dyes [71,72]. Considering all the disadvantages of the small molecule dyes such as cell toxicity, cell unspecific loading, cell internalization, *etc*. it would be advantageous to perform these measurements with tissue-specific targeted GEVIs. This would enable researchers to perform *in situ* recordings, as done for genetically encoded calcium sensors [73].

The first optical mapping of the heart with GEVIs *in vivo* was reported for zebrafish using Arch(D95N) as part of a dual function calcium and voltage reporter (CaViar) [41]. In this paper, optical mapping of action potentials and calcium transients in combination with pharmacological probing documented the chamber specific developmental transition in ionic currents [41].

**Figure 2:**
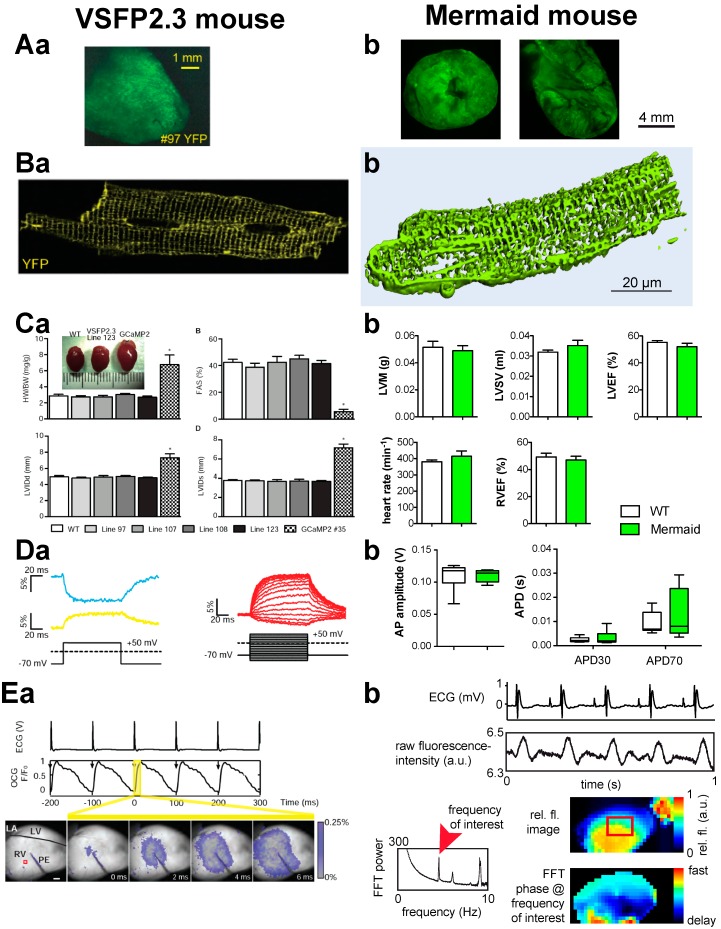
Transgenic mice expressing a Genetically Encoded Voltage Indicator (GEVI) for optical mapping of the heart. Comparison of mice expressing VSFP2.3 (left, all panels (**a**)) and Mermaid (right, all panels (**b**)). Although slightly different parameters are presented, both mice show consistent data. (**A**) Cardiac appearance of the GEVI expression. (**a**) View of the excised heart; (**b**) Cut open heart: left, short axis; right, long axis; (**B**) Isolated cells expressing the GEVI on the plasmalemma, including T-tubules. (**a**) Confocal section; (**b**) 3D reconstruction based on confocal recordings; (**C**) GEVIs neither alter morphologic nor functional cardiac parameters. (**a**) Echocardiographic based parameters of different VSFP2.3 mouse lines compared to WT and GCaMP2 mice [73]: top left, heart weight to body weight ratio (HW/BW); top right, fractional area shortening (FAS); bottom left, diastolic left ventricular inner diameter (LVIDd); bottom right, systolic left ventricular inner diameter (LVIDs). None of the mice lines showed any significant differences except for the comparison with GCaMP2 mice (*n* = 8 mice per genotype); (**b**) Magnetic resonance imaging based parameters of Mermaid mice compared to WT: top left, left ventricular mass (LVM); top middle, left ventricular stroke volume (LVSV); top right, left ventricular ejection fraction (LVEF); bottom left, heart rate; bottom middle, right ventricular ejection fraction (RVEF). None of the parameters showed significant differences between Mermaid and WT mice (*n* = 6 mice per genotype); (**D**) Patch-clamp related measurements in mice expressing GEVI. (**a**) Left: representative traces of CFP and YFP in response to a voltage step from −70 to +50 mV in cardiomyocytes expressing VSFP2.3. Right: YFP/CFP ratios in response to a voltage protocol as depicted in cardiomyocytes expressing VSFP2.3, the optical signals show a pronounced delay compared to the command voltage as was also shown for Mermaid in cardiomyocytes [33]; (**b**) Action potential (AP) properties of Mermaid mice compared to WT at a stimulation frequency of 5 Hz: left, AP amplitude; right, AP duration (APD) for 30% and 70% repolarization. None of the parameters showed significant differences between Mermaid and WT mice (*n* = 10 cells per genotype); (**E**) Proof-of-principle for Langendorff-perfused heart recordings of mice expressing GEVI. (**a**) Synchronous electrical cardiograms (ECG) and optical cardiograms (OCG) supplemented with representative images during 10 Hz electrical pacing via a point electrode; (**b**) Synchronous ECG and raw fluorescence traces (based on the region of interest as indicated by the red rectangle in the relative fluorescence (rel. fl.) image) of an autonomous beating heart (top traces) were subjected to a Fast Fourier Transformation (FFT, left graph). The FFT phase at the frequency of interest (beating frequency of the heart) was visualized for each pixel (bottom right) to map the temporal AP distribution over the heart. Items in the left column (all panels (**a**)) are reproduced from [31], with permission from Wolters Kluwer.

Recently, a report based on a transgenic mice line expressing VSFP2.3 introduced the methodology to mammals [31]. As depicted in Figure 2, these published results are in agreement with our own observations based on a transgenic mouse expressing Mermaid. Both approaches show a homogenous expression in the heart (Figure 2A), the right subcellular localization at the plasma membrane (Figure 2B), normal development and function of the heart (Figure 2C), undisturbed action potentials in agreement with patch-clamp (Figure 2D) and an optical read out of the cardiac action potential (Figure 2E). However, a limitation is the minute signal change (max. 0.25%, Figure 2E). It is worthwhile to highlight that the examples shown in Figure 2 provide a first proof that optical mapping based on GEVIs is possible in mammalian hearts, but routine measurements to investigate putative differences under different experimental conditions requires improved sensors and experimental settings.

## 4. Summary and Perspective

The development of GEVIs is not yet as mature as genetically encoded calcium indicators (GECIs) [74]. Both sensor types are related to one another in circulation research trough the process of excitation-contraction coupling [75]. However, GEVI design and characterization has gained large momentum in recent years resulting in an exponential increase in the numbers of publications. The major improvements of the sensors are accompanied by an increasing number of scientists recognizing the tremendous potential of such genetically encoded probes. Thus, recent papers on GEVIs started broadening focus from the engineering, characterization and proof of principle to reports of their application in physiology or pathophysiology-driven studies [37,76].

Although the development of the GEVIs was initially led by applications in neuroscience [29], the examples presented here on cardiac cells and cardiac tissues pave the way for an application in circulation research as well as in the pharmaceutical industry, especially for cardiac safety screens. We face the situation of a delay between the introduction of a GEVIs and their application, because of intermediate steps, including the generation of transgenic animals or viruses for gene transfer [77] and the establishment of a reproducible and robust read-out mode. In this context, we can expect that recently introduced GEVIs (see lower part of Table 1) and novel sensors to be developed will replace the GEVIs so far used in circulation research.

Future sensors will further drive forward the unifying advantageous properties of single GEVIs. This will enable superior properties of GEVIs, such as a combination of high fluorescence intensity and high dynamic range, which will allow applications in non-excitable cells. Red or far-red GEVIs will allow measurements combining several sensors, e.g., phosphorylation probes [78] in combination with GEVIs, or probing red blood cells, where quantitative biosensors need to be outside the absorption spectrum of hemoglobin [79]. The combination of high intensity, high dynamic range and high temporal response will facilitate investigations of sub-cellular components of action potentials as already performed for calcium transients and thus reveal inhomogeneous generation of voltage signals or inhomogeneous distribution and propagation of voltage changes that might contribute to, e.g., cardiac alternans or other forms of arrhythmias in the heart [80].

With these improvements, the general applicability of GEVIs will rise and render it a powerful extension of traditional electrophysiology. The latest progress in both GEVI development and imaging technology may bring optogenetic readouts more in line with classical current-clamp measurements and may for particular applications such as those described above even outperform them. This may especially hold true in combination with optogenetic induction of action potentials using channelrhodopsin or related proteins [36].
